# Regulation of Ca_V_1.3 Ca^2+^ channels in cochlear inner hair cells

**DOI:** 10.1186/2050-6511-13-S1-A49

**Published:** 2012-09-17

**Authors:** Alexandra Pinggera, Niels Brandt, Gurjot Kaur, Jutta Engel, Jörg Striessnig

**Affiliations:** 1 Department of Pharmacology and Toxicology, Institute of Pharmacy, Center for Molecular Biosciences Innsbruck, University of Innsbruck, 6020 Innsbruck, Austria; 2 Department of Biophysics, Saarland University, 66424 Homburg, Germany

## Background

Ca_V_1.3 Ca^2+^ channels are voltage-gated L-type calcium channels, regulating many different physiological functions including neurotransmitter release in cochlear inner hair cells (IHCs) after sound-evoked stimulation. In IHCs, the Ca_V_1.3 channel shows rapid activation after stimulation with very slow inactivation kinetics, whereas in other tissues (heart and brain) the channel underlies a very strong inactivation. The exact mechanism for the very slow inactivation observed in IHCs is not known so far. Interaction of the auxiliary β subunit with RIM2α, an active zone scaffolding protein, slows down calcium-dependent inactivation (CDI) and voltage-dependent inactivation (VDI); however, it does not fully account for the even slower inactivation kinetics observed in IHCs, as recently published by our lab. RIM binding proteins (RBPs), another group of active zone proteins, are known to interact with RIM and with the Ca_V_1.3 α1 subunit C-terminus. We therefore hypothesized that interaction of the Ca^2+^ channel with both RIM and RBPs results in a large signaling complex that restrains gating transitions, leading to the slow inactivation kinetics of the Ca_V_1.3 channel in IHCs.

## Methods

In order to investigate whether RBP isoforms and RIM proteins are expressed in inner hair cells, we performed nested PCR. Complex formation of RIM and RBPs with the channel after co-expression in a recombinant system was demonstrated by immunofluorescence microscopy.

## Results

So far we could detect RIM2α, RBP2 and RBP3 transcripts in immature and mature IHCs with nested PCR. Preliminary immunofluorescence data also show that RIM and RBPs form a complex that binds to channel-derived peptides.

## Conclusions

Our results are in agreement with the hypothesis, justifying further experiments. Therefore we will show the co-localization of Ca_V_1.3, RIM and RBP in ribbon synapses of IHCs (immunohistochemistry) and study the functional consequences of such complex formation by patch clamp recordings.

